# Genetic variation in Dip5, an amino acid permease, and Pdr5, a multiple drug transporter, regulates glyphosate resistance in *S*. *cerevisiae*

**DOI:** 10.1371/journal.pone.0187522

**Published:** 2017-11-20

**Authors:** Xiaoqing Rong-Mullins, Apoorva Ravishankar, Kirsten A. McNeal, Zachery R. Lonergan, Audrey C. Biega, J. Philip Creamer, Jennifer E. G. Gallagher

**Affiliations:** Department of Biology, West Virginia University, Morgantown, West Virginia, United States of America; University of Leicester, UNITED KINGDOM

## Abstract

*S*. *cerevisiae* from different environments are subject to a wide range of selective pressures, whether intentional or by happenstance. Chemicals classified by their application, such as herbicides, fungicides and antibiotics, can affect non-target organisms. First marketed as RoundUp^™^, glyphosate is the most widely used herbicide. In plants, glyphosate inhibits EPSPS, of the shikimate pathway, which is present in many organisms but lacking in mammals. The shikimate pathway produces chorismate which is the precursor to all the aromatic amino acids, *para*-aminobenzoic acid, and Coenzyme Q10. Crops engineered to be resistant to glyphosate contain a homolog of EPSPS that is not bound by glyphosate. Here, we show that *S*. *cerevisiae* has a wide-range of glyphosate resistance. Sequence comparison between the target proteins, i.e., the plant EPSPS and the yeast orthologous protein Aro1, predicted that yeast would be resistant to glyphosate. However, the growth variation seen in the subset of yeast tested was not due to polymorphisms within Aro1, instead, it was caused by genetic variation in an ABC multiple drug transporter, Pdr5, and an amino acid permease, Dip5. Using genetic variation as a probe into glyphosate response, we uncovered mechanisms that contribute to the transportation of glyphosate in and out of the cell. Taking advantage of the natural genetic variation within yeast and measuring growth under different conditions that would change the use of the shikimate pathway, we uncovered a general transport mechanism of glyphosate into eukaryotic cells.

## Introduction

**Fig 1:**
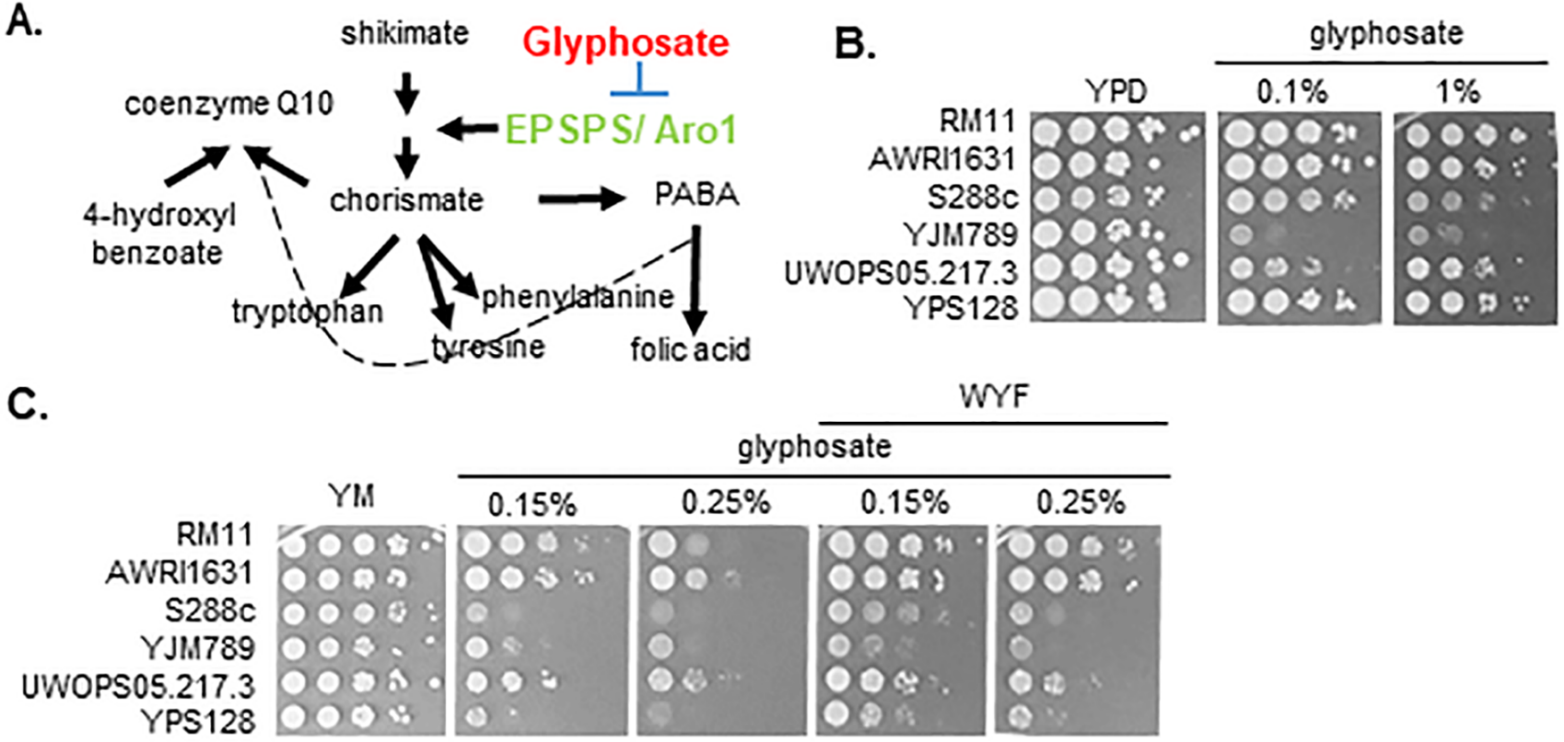
Genetic variation effects growth inhibition by glyphosate. **A**. Shikimate pathway produces the precursor for phenylalanine, tyrosine, tryptophan, *para*-Aminobenzoic acid (PABA), folic acid and Coenzyme Q10. The canonical target of glyphosate is EPSPS in plants and Aro1 is the yeast homolog of EPSPS. PABA and 4-hydroxylbenzoate can be converted to Coenzyme Q10. **B**. Serial dilution of genetically diverse yeast on rich media (YPD) with dilutions of glyphosate as indicated (1% vol/ vol is equivalent to 78 mM). **C**. Serial dilution of genetically diverse yeast on minimal media with glyphosate. Aromatic amino acids, tryptophan (W), tyrosine (Y) and phenylalanine (F) were added to YM plates to make WYF.

RoundUp^™^ is a non-selective herbicide containing glyphosate and a variety of additives such as detergents. As a broad-spectrum herbicide, glyphosate, the active ingredient in RoundUp^™^, inhibits production of chorismate, the precursor for tryptophan, tyrosine, phenylalanine, Coenzyme Q10, and *para*-Aminobenzoic acid (PABA), and its supplementation circumvents this growth inhibition in plants [1]. Glyphosate inhibits 5-enolpyruvylshikimate-3-phosphate synthase (EPSPS) in plants, an enzyme of the shikimate pathway (Fig 1A). Sensitive alleles of EPSPS are directly bound by glyphosate [2, 3]. Changing amino acids in the glyphosate binding site of EPSPS or overexpression of EPSPS confers RoundUp^™^ resistance [4, 5]. The functional ortholog of EPSPS in yeast is Aro1 and it contains additional enzymatic functions (S1A Fig) that are encoded by separate proteins in plants and bacteria [6].

Relatively low human acute toxicity and the broad range of susceptible plants has encouraged widespread use of glyphosate, and its effects on non-target organisms are becoming pervasive [7]. Humans do not have EPSPS and cannot make aromatic compounds, hence acquire these essential nutrients through their diet or microbiota [8, 9]. The few reports of acute glyphosate toxicity in humans are likely from the detergents that are part of the commercial formulations [10]. Spraying glyphosate on crops and other weeds also exposes nearby organisms to glyphosate, including insects, bacteria and fungi. *Saccharomyces cerevisiae*, a species with tremendous genetic diversity, occupies a wide range of niches, making it well suited for investigations of adaptation to new environmental stressors. There is more genetic diversity (SNPs/ Kb) between two different strains of yeast than among the entire human species [11–14]. There are 60,000 SNPs in the 12 Mb yeast genome [15] and 10 million SNPs in the 3,200 Mb human genome. [16]. Therefore, yeast is an ideal model organism to address acquisition of glyphosate resistance by examining variable alleles.

Two different mechanisms of glyphosate resistance have been uncovered in plants and bacteria. The first mechanism involves changing the ability of EPSPS to be bound by glyphosate, either by mutations that alter glyphosate binding efficacy (reviewed in [17]) or amplification of the EPSPS gene. The second mechanism involves increased levels of transport pathway elements such as ABC transporters, that move it to the vacuole and in turn neutralize the effect of glyphosate [18]. Pdr5 is an ABC transporter that is orthologous to the human Mdr1 and is often amplified in chemotherapeutic resistant cancers [19].

To determine if there were other underlying mechanisms of tolerance, in this study we characterized the yeast response to glyphosate. In different growth conditions, yeast exhibited a wide range of growth inhibition by glyphosate. The only known target of glyphosate is Aro1; however, polymorphisms in Aro1 were not responsible for the genetic variation of growth inhibition by glyphosate. Supplementing aromatic amino acids, which should bypass the shikimate pathway, improved growth in a dose-dependent manner but there was still variation in growth. To address the media-dependent genetic variation in glyphosate tolerance, Quantitative Trait Loci (QTL) analysis was carried out between two strains that demonstrated the greatest divergence in phenotypic response to glyphosate. In minimal media, the variation in glyphosate resistance was mapped to an amino acid permease, Dip5. While *DIP5* deletion increased glyphosate resistance, expression of the resistant allele further improved growth of yeast on glyphosate which suggested that the resistant allele has additional functions. Dip5 function was decreased by the addition of aspartic acid, resulting in relieved growth inhibition of all yeast tested in response to glyphosate. However, the magnitude of increased *DIP5* mRNA levels in yeast that were treated with glyphosate did not correlate with changes in growth across strains. Together this suggests that, regardless of the genetic variation in *DIP5* among the various yeast strains, the Dip5 protein was similarly regulated by aspartic and glutamic acid. Dip5 can import glyphosate into the yeast cell and the resistant allele has additional functions that the sensitive allele lacks in response to glyphosate. In addition, variation in glyphosate resistance in rich media was mapped to an ABC pleiotropic drug transporter, Pdr5. Deletion of *PDR5* resulted in the loss of glyphosate tolerance, while expression of the resistant allele conferred glyphosate resistance and expression of the sensitive allele did not. While it is likely that there are many proteins that regulate the response to and the transport of glyphosate, this study sought to identify the divergent genes between the two strains.

## Results

There is a tremendous amount of phenotypic variation among yeast strains in response to different forms of stress. As rich media (YPD) contains all the required amino acids, it represses amino acid biosynthetic pathways and permits yeast to transport amino acids from the media into the cell. Six genetically diverse yeast isolated from different environments were grown on YPD supplemented with glyphosate (Fig 1B), RM11(a wine yard isolate), AWRI1631 (used for commercial wine making), S288c (a laboratory strain), YJM789 (a clinical isolate), UWOPS05.217.3 (isolated from bertam palm nectar), and YPS128 (isolated from the soil under an oak tree). These strains showed little change in growth on solid media in the presence of 0.1% volume/volume glyphosate (78 mM) on YPD. The growth of YJM789, a clinical isolate, was reduced by the addition of 0.1% glyphosate in YPD (Fig 1B). At levels ten times higher, the growth of YJM789 was nearly completely inhibited and the growth of S288c was slightly reduced. The commercial preparation of glyphosate contains additives such as detergents to increase the tissue penetration of glyphosate. To better reflect conditions that organisms would be exposed to, we chose to use the commercial preparation of glyphosate (RoundUp^™^) rather than pure glyphosate. In minimal media (YM), yeast were required to make all amino acids using ammonium sulfate as the sole nitrogen source. Under this growth condition, S288c, YJM789, and YPS128 were sensitive to 0.15% glyphosate and all the strains tested, grew more slowly on 0.25% glyphosate treatment (Fig 1C). AWRI1631, RM11, and UWOPS05.217.3, which are agricultural isolates, demonstrated higher glyphosate tolerance in YM. Hence, among the agricultural isolates RM11 was found to be the most resistant to glyphosate exposure, followed by AWRI1631 and UWOPS05.217.3. Whereas, YJM789 was the most sensitive followed by YPS128 and S288c. When aromatic amino acids (tryptophan (W), tyrosine (Y), and phenylalanine (F)) were added back to YM (WYF), growth inhibition of S288c and YPS128 were alleviated (Fig 1C) and the growth of YJM789 showed improvement at lower glyphosate levels.

**Fig 2:**
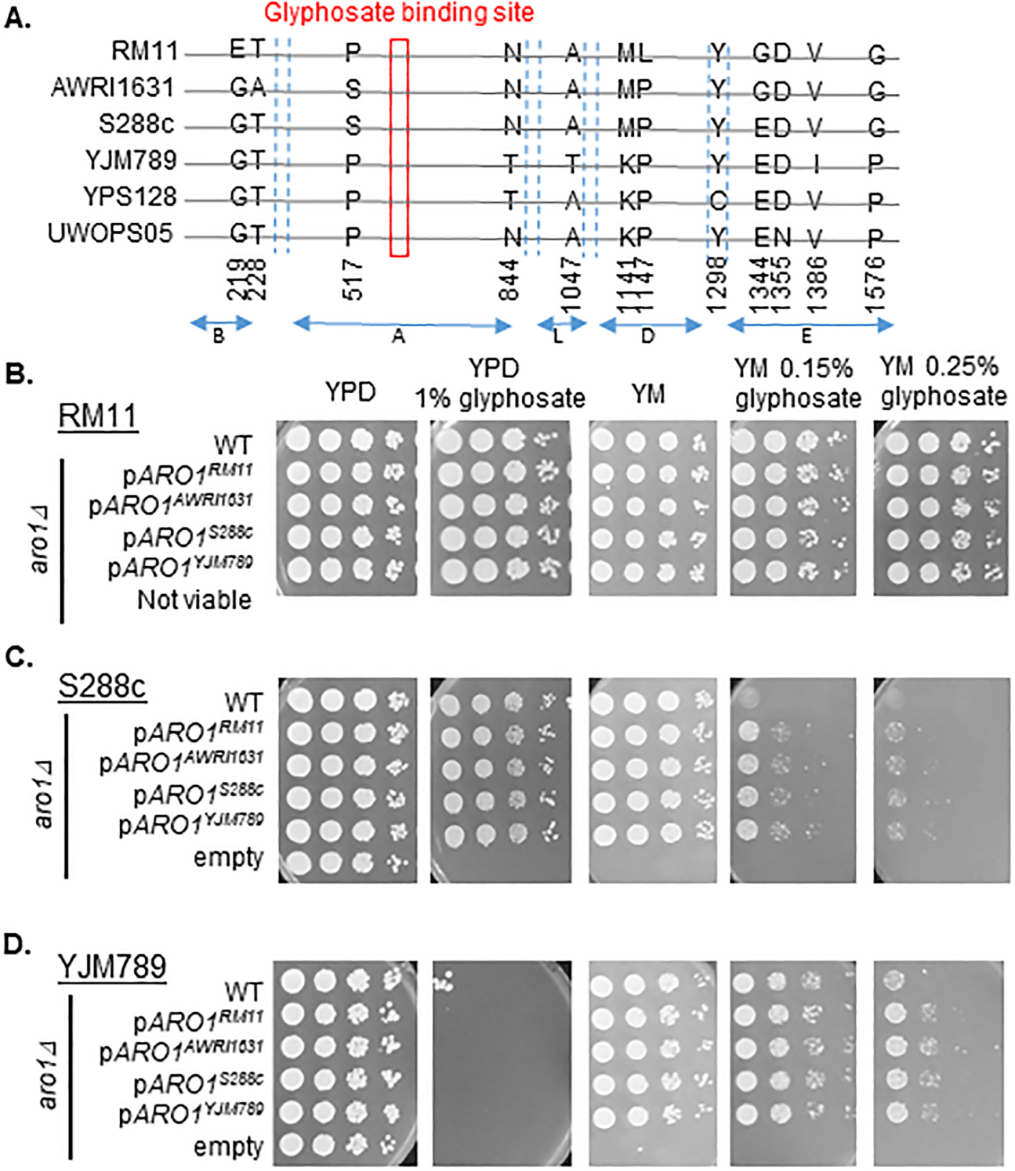
Contribution of the genetic variation within Aro1 to glyphosate resistance. **A**. Protein alignment of Aro1 from genetically diverse yeast strains. **B**. Serial dilutions of haploid *aro1Δ* in RM11, **C**. S288c, and **D**. YJM789, expressing different alleles of plasmid encoded *ARO1* were grown on YPD and YM with and without glyphosate. Rows labeled empty have pGS36 plasmid with no *ARO1*. Parental strains with the endogenous *ARO1* expressed from the chromosome labeled WT carry an empty plasmid (pGS36).

Numerous polymorphisms in the plant and bacterial homologs of Aro1 result in glyphosate resistance [17]. We predicted that yeast would be resistant to RoundUp^™^ by comparing mutations that have been mapped in AroA from *E*. *coli* and the yeast amino acid sequence of Aro1 [4, 20, 21] (S1B Fig). Yet, different strains of yeast showed differences in sensitivity to glyphosate that could be rescued with the supplementation of WYF. The polymorphisms in Aro1 across these six different yeast strains were outside the glyphosate binding domain (Fig 2A). To determine if polymorphisms in Aro1 contributed to the variation in glyphosate resistance, *ARO1* was knocked out. The RM11heterozygous *aro1Δ/ ARO1* strain was sporulated and the tetrads were dissected onto YPD. A lethal mutation segregated in a 2:0 pattern that was linked to Nat^R^ in two independent knockouts, i.e., the *aro1Δ* was inviable in RM11 (S1D Fig). *ARO1* was successfully knockout in S288c and YJM789 haploid yeast and could grow if WYF was supplemented (Fig 2).

To determine if *ARO1* was an essential gene in RM11 or if RM11 contained an allele that was synthetically lethal in combination with an *aro1* deletion, RM11 (wild-type) was crossed with S288c *aro1Δ* (viable mutant), sporulated and dissected onto YPD (S1E Fig). Fourteen tetrads were dissected and 34 spores were viable. Twelve viable *aro1Δ* segregants from the F1 hybrids ruled out the possibility that Aro1^RM11^ gained an essential function, but instead *aro1Δ* was synthetically lethal with an unknown allele present in the RM11 genome and not present in the S288c genome. The unknown allele was found to be unlinked to the size of the colony, the *MAT*, *HO*, and *ARO1* loci. In conclusion, the Aro1 deletion in most strains was found to be viable, with an exception of RM11.

To assess the impact of the genetic variation of Aro1 in response to glyphosate, four different alleles of *ARO1* were cloned under their endogenous promoter and terminator into plasmids and transformed into *aro1Δ* yeast. *ARO1* from AWRI1631 and RM11 represent alleles that are present in glyphosate resistant yeast, while *ARO1* from S288c and YJM789 are alleles from less tolerant strains. While *ARO1* was essential in RM11, plasmids encoding alleles of *ARO1* were transformed into the heterozygous diploid, and the haploid knockouts with the plasmid were recovered after sporulation (Fig 2B). S288c *aro1Δ* and YJM789 *aro1Δ* were viable on YPD and WYF but not on YM (Fig 2C and 2D). It can be concluded that all alleles of *ARO1* could complement *aro1Δ* mutation in RM11, S288c and YJM789 yeast, because the *aro1Δ* yeast with plasmid expressed *ARO1* grew on YM (Fig 2) to similar levels as the wild-type parents carrying an empty plasmid. When grown on YM with glyphosate, yeast cells showed no difference in growth irrespective of the *ARO1* allele expressed. However, S288c and YJM789 with *ARO1* expressed from the plasmid consistently grew slightly better than yeast expressing chromosomal *ARO1*. The mRNA levels of *ARO1* from RM11, YJM789 and YJM789 *aro1Δ* were quantified. In YJM789, *ARO1* expressed from both the chromosomal location and from a plasmid was approximately the same (S1C Fig). There was a slight decrease in the levels of *ARO1* mRNA found when YJM789 was exposed to glyphosate. In YJM789, expression of *ARO1*^*RM11*^ was no different from *ARO1*^*YJM798*^. Yet, there was an increase in *ARO1* mRNA levels in RM11 compared to YJM789 in untreated growth conditions.

**Fig 3:**
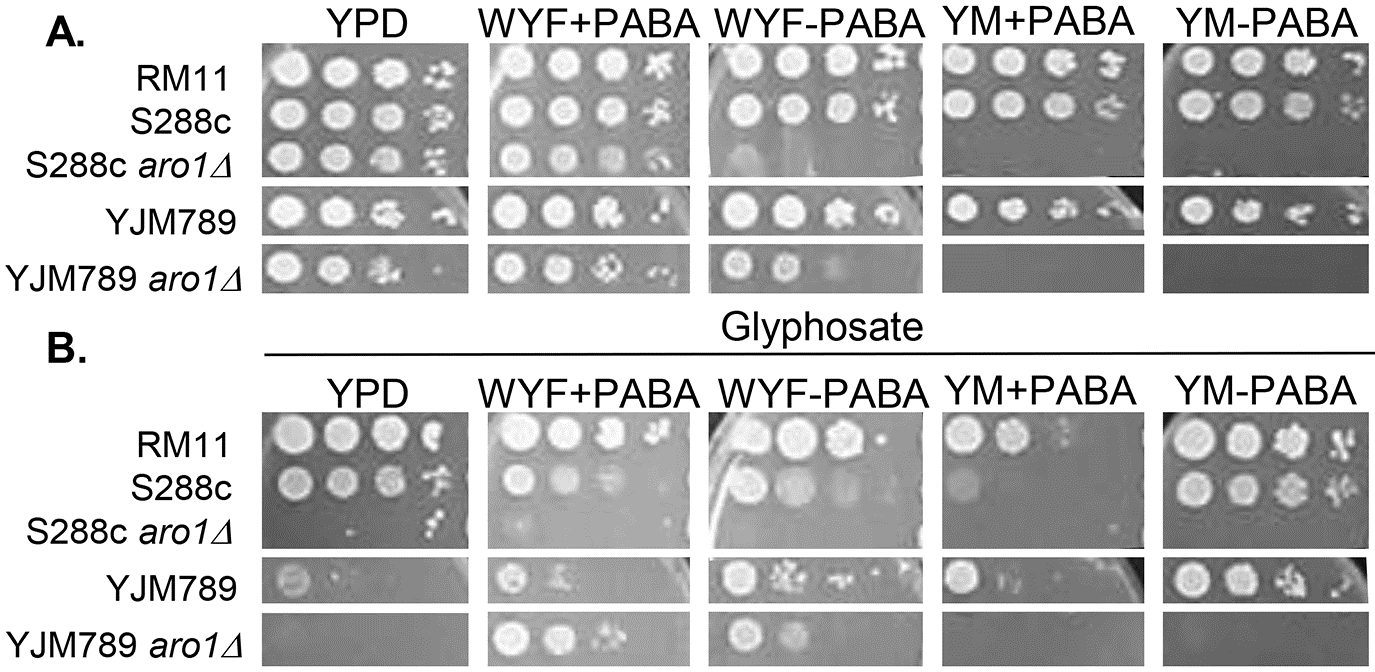
**A. Different responses of *aro1****Δ* **yeast on media supplemented with different aromatic metabolites**. Wild-type RM11 was compared to S288c (GSY147) and YJM789 with and without *ARO1*, three days in minimal media supplemented with aromatic amino acids (+WYF) or *para*-aminobenzoic acid (+PABA) or without these metabolites (-WYF or -PABA). **B**. Different responses of *aro1Δ* yeast on glyphosate. Wild-type RM11 was compared to S288c (GSY147) and YJM789 with and without *ARO1* on 0.15% glyphosate three days in minimal media supplemented with +WYF or +PABA or without these metabolites (-WYF or -PABA).

The primary target of glyphosate is inhibition of Aro1 in the chorismate pathway and the genetic variation resulting in growth inhibition in response to glyphosate, was not due to genetic differences in Aro1. Therefore, the genetic variation in response to glyphosate could be due to genetic variation in other components of the chorismate pathway or in an unrelated pathway. To explore the differences in the chorismate pathway between S288c and YJM789 yeast, the *aro1* knockouts were tested on various media. Chorismate is the precursor for aromatic amino acids, PABA, and Coenzyme Q10 (CoQ10). *para*-Aminobenzoic acid (PABA) is essential for the production of folic acid derivatives and can be converted to Coenzyme Q10 precursor (Fig 1A). CoQ10 is involved in respiration and is not essential for yeast viability. The commercial preparation of YM contains PABA and folate [22] and is noted here as YM+PABA. S288c *aro1Δ* could not grow on WYF-PABA (YM without PABA, supplemented with WYF), while the wild-type parent could grow. In YJM789 *aro1Δ*, growth was only slightly reduced on WYF-PABA as compared to its wild-type parent; this difference in growth between S288c *aro1Δ* and YJM789 *aro1Δ* suggested the existence of an alternative pathway present in YJM789 that is absent in S288c. YJM789 was able to convert WYF to PABA and influence cell growth. RM11 required *ARO1* for viability and therefore the knockout could not be tested (Fig 3A). Additionally, PABA-free, and folate-free media was tested, where no change in growth was seen in comparison to PABA-free media, and thus this avenue was not further examined.

To determine if supplementation of yeast with downstream metabolites synthesized from chorismate could bypass the growth inhibition in the presence of glyphosate, yeast cells were grown on media supplemented with WYF in either the presence or absence of PABA. To assess whether PABA had a role in glyphosate growth inhibition, yeast cells were also tested on PABA-free media, supplemented with WYF. S288c *aro1Δ* were more sensitive than the wild-type parent to glyphosate on WYF media (Fig 3B), while YJM789 *aro1Δ* was more resistant to glyphosate than the wild-type parent. Only growth of RM11 on YM with glyphosate showed a slight decrease, while no change was detected when RM11 was grown in other conditions.

**Fig 4:**
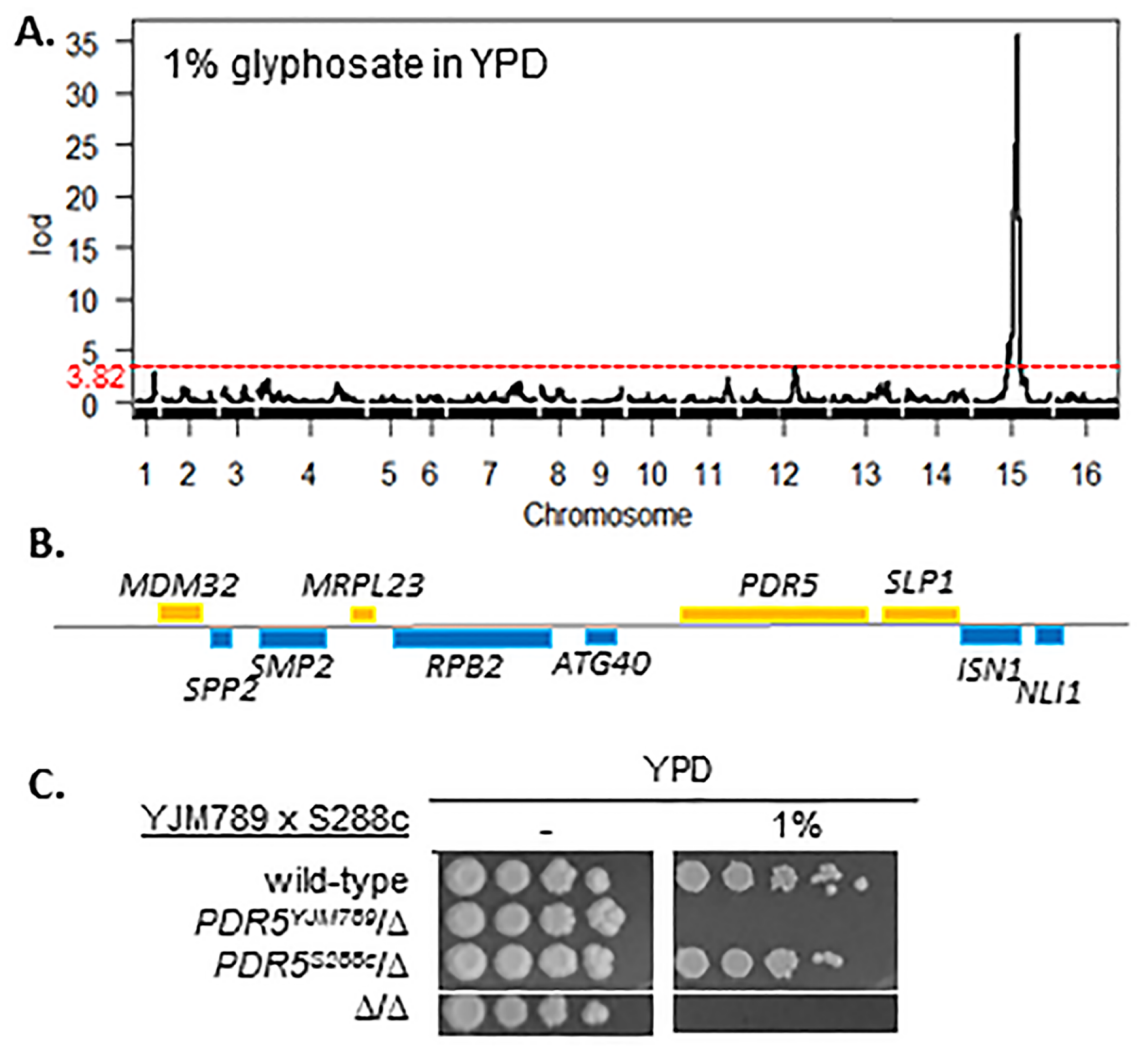
Genetic linkage analysis of glyphosate sensitivity in glyphosate on rich media. **A**. Genetic linkage of sensitivity of YJM789 to 1% glyphosate in YPD. LOD score (y-axis) was mapped across the yeast genome (x-axis) with chromosomes numbered left to right. The LOD significant levels (alpha = 0.05) was 3.82 and was marked by a red dashed line. **B**. The genomic loci under the peak located on chromosome 15 contains 10 genes. Genes encoded on the top strand are in yellow and genes encoded on the bottom strand are blue. **C**. Serial dilution of S288c (GSY147) x YJM789 hybrids in which the entire *PDR5* coding region in either parent was deleted (Δ), crossed, and the resulting hemizygotes were grown on rich media with and without 1% of glyphosate.

While genetic variation leads to differences in glyphosate resistance, the phenotypic response to glyphosate was not due to variation in *ARO1*. We chose to map the genes associated with glyphosate resistance in S288c and YJM789 because of the variation in their growth in YPD, WYF and YM with glyphosate. We tested the growth of 125 recombinant haploid segregants from a hybrid of S288c and YJM789 [23]. For S288c in YPD with 1% glyphosate, one locus of interest was identified on chromosome 15 with a LOD score of 35.5 (Fig 4A). *PDR5*, an ABC transporter that confers resistance to a wide-range of structurally diverse chemicals [24, 25] was located within this region (Fig 4B). *PDR5* has previously been implicated in plant response to glyphosate [18]. The role of each allele of *PDR5* was tested in YJM789 and S288c diploid hybrids. *PDR5* was knocked out from each parent and mated with the other parent. The resulting hemizygous strains are identical except for the *PDR5* allele that the hybrid inherited from one parent (Fig 4C). The *PDR5*^*YJM789*^*/Δ* hybrid was sensitive to glyphosate on YPD, while the *PDR5*^*S288c*^*/Δ* hybrid demonstrated the same level of tolerance as that of the wild-type hybrid. The sensitivity of the *PDR5*^*YJM789*^*/Δ* hybrid was similar to that of the homozygous knockout mutant. Pdr5 is a highly polymorphic protein, where the hyper-variability of its genetic sequence allows for greater specificity and tolerance to a wide array of chemicals per allele [26]. Between YJM789 and S288c, there is a 5% difference in amino acid sequence. Among twelve available sequences that were analyzed, no obvious polymorphism was found in common among the yeast that were sensitive to glyphosate on YPD (S2 Fig).

**Fig 5:**
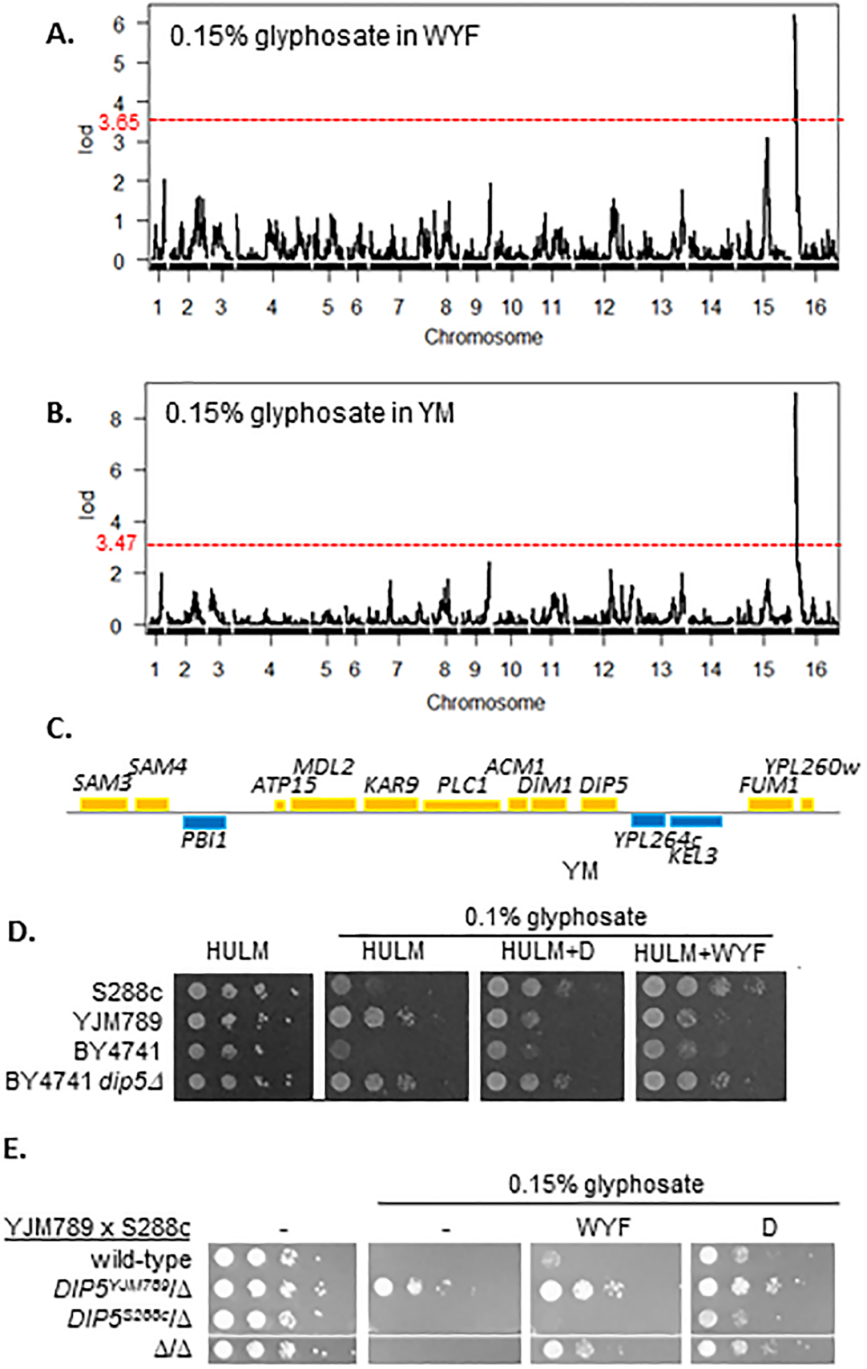
Genetic Linkage analysis of glyphosate sensitivity in glyphosate on minimal media with and without aromatic amino acids. **A**. Genetic linkage of sensitivity of YJM789 to 0.15% glyphosate in yeast minimal media supplemented with aromatic amino acids (WYF). LOD score (y-axis) was mapped across the yeast genome (x-axis). The LOD significant levels (alpha = 0.05) was 3.65 and was marked by a red dashed line. **B**. Genetic linkage of sensitivity of YJM789 to 0.15% glyphosate in yeast minimal media (YM). LOD score (y-axis) was mapped across the yeast genome (x-axis). The LOD significant levels (alpha = 0.05) was 3.47 and was marked by a red dashed line. **C**. The genomic loci under the peak located on chromosome 16 contains 14 genes. Genes encoded on the top strand are in yellow and genes encoded on the bottom strand are blue. **D**. Serial dilutions of S288c (GSY147), YJM789 and BY4741 with *DIP5* knocked out grown on YM (HULM), WYF, aspartic acid (D) with glyphosate at the concentrations indicated. Histidine, uracil, leucine and methionine (HULM) were supplemented for growth of BY4741. **E**. Serial dilutions of S288c (GSY147) x YJM789 hybrids. The entire *DIP5* coding region in either parent was deleted (Δ), crossed, and the resulting hemizygotes were grown on solid media YM, WYF, aspartic acid (D) with glyphosate at the indicated concentrations and supplemented.

Among the S288c and YJM789 segregants grown on WYF, an additional peak on chromosome 16 was found to be linked to glyphosate response (Fig 5A). The aforementioned peak on chromosome 15 associated with *PDR5* in YPD was observed, however the peak fell below the level of significance (LOD < 3.65) in YM with WYF. Additionally, the same peak on chromosome 16 was also identified when yeast were grown on YM without WYF supplementation (Fig 5B). Fourteen candidate genes were in the region under this peak (Fig 5C) and were evaluated using the yeast knockout collection, where the likely candidate was *DIP5* [27]. The knockout collection was constructed in an S288c related strain, BY4741 that has multiple amino acid auxotrophies and several other differences from GSY147 (another S288c related strain) [28]. To circumvent the amino acid auxotrophies and achieve normal growth, BY4741 was supplemented with histidine, uracil, methionine, and leucine (HULM). BY4741 is highly sensitive to glyphosate and to accommodate for this, the level of glyphosate was reduced to 0.1%. This reduction allowed for a more pronounced rescue of BY4741 *dip5Δ*.

Dip5 is a high affinity permease for aspartic and glutamic acid [29] and when these amino acids are in excess, the localization of the protein at the plasma membrane is reduced [30]. To assess if down-regulation of Dip5 at the plasma membrane could rescue glyphosate-induced growth inhibition, cells were grown in glyphosate media supplemented with aspartic acid. Aspartic acid (D) rescue was similar to that of WYF rescue and was specific. Whereas, the addition of other amino acids (HULM) did not rescue glyphosate sensitive yeast (Fig 5D). Aspartic acid is not a product of the shikimate pathway. Hence, the increased glyphosate tolerance of yeast when supplemented with aspartic acid suggests that glyphosate was imported into yeast via Dip5, where the increased tolerance is achieved through aspartic acid-mediated downregulation of Dip5. The role of each allele of *DIP5* was tested in reciprocal hemizygous YJM789 and S288c diploid hybrids. *DIP5* was knocked out from each parent, which was then mated with the other parent. The resulting hemizygous strains are identical except for the allele of *DIP5* the hybrid inherited from one parent (Fig 5E). The wildtype hybrid, the homozygous mutant, and *DIP5*^*S288c*^*/Δ* hybrid were sensitive to glyphosate on minimal media, while the *DIP5*^*YJM789*^*/Δ* hybrid was resistant. The addition of WYF, supplements amino acids from the shikimate pathway. With 0.15% glyphosate, only the homozygous mutant diploid hybrid could be rescued by the addition of WYF. However, the addition of aspartic acid recused all strains.

**Fig 6:**
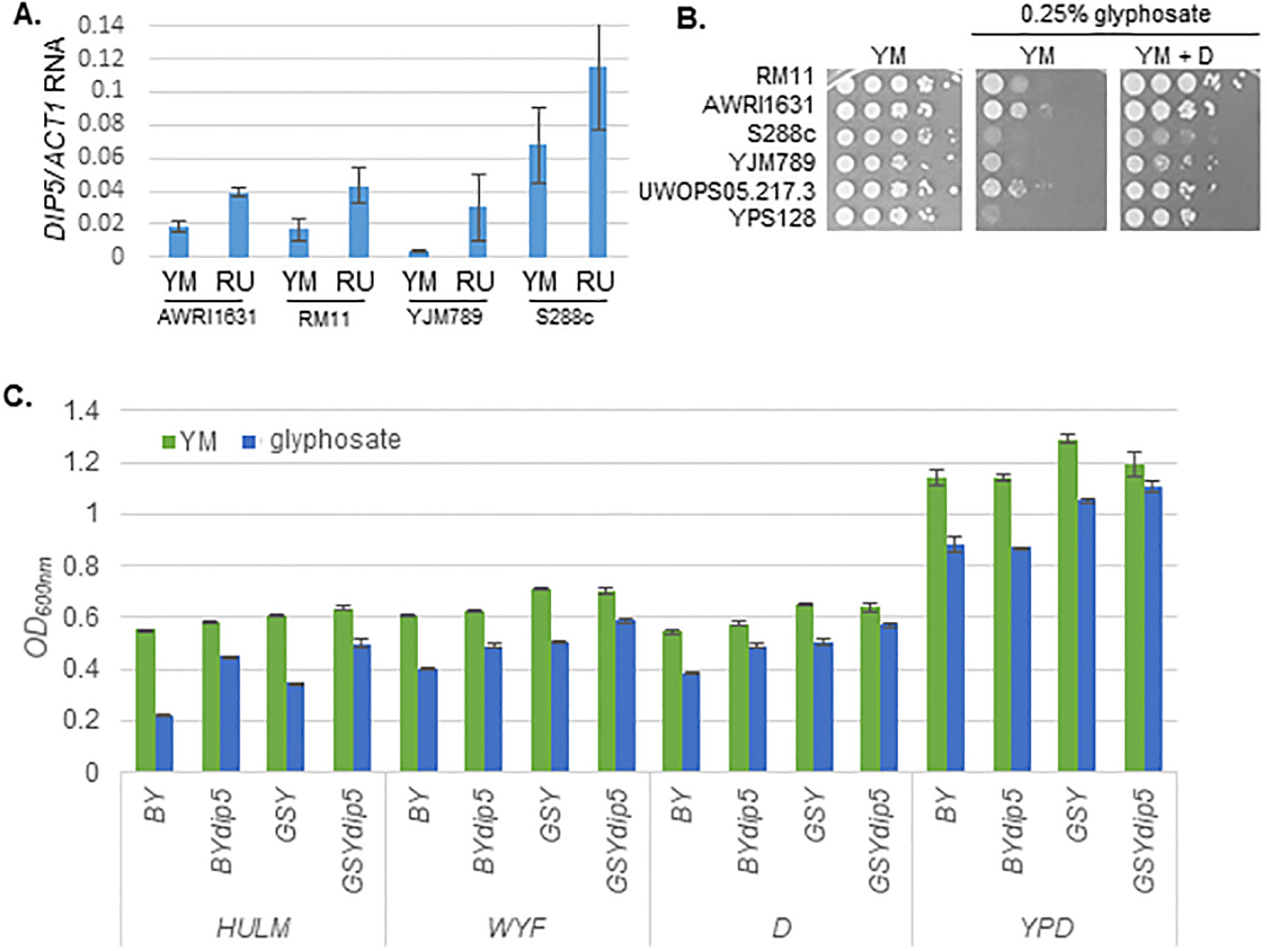
Regulation of *DIP5* by glyphosate and aspartic acid. **A**. RNA expression levels of *DIP5* mRNA in AWRI1631, RM11, YJM789 and S288c grown in YM with and without 0.25% glyphosate (RU). Q RT-PCR of *DIP5* mRNA levels are normalized to *ACT1* mRNA. **B**. Serial dilution of genetically diverse yeast on minimal media with glyphosate supplemented with aspartic acid. **C**. Different responses of *dip5Δ* yeast in liquid media supplemented with different aromatic metabolites and amino acids on exposure to glyphosate. BY4741 and GSY147 were grown in the presence of glyphosate (0.1% in HULM, WYF and D and 1% in YPD) and the optical density was measured in log phase (10 hr).

As in the case of Pdr5, we could not identify a single polymorphism that was associated with all the resistant strains. However, as the presence of the *DIP5*^*S288c*^ allele decreased glyphosate resistance while presence of the *DIP5*^*YJM789*^ conferred tolerance, the responsible polymorphism could be contributing to a hypomorphy-induced resistance—such as a mutation in the promoter sequence resulting in decreased expression of *DIP5*. This hypomorphy could be suspected to be responsible for the resistance of the YJM789 *DIP5* allele, as *DIP5* is expressed in levels greater than two-fold higher in S288c yeast as compared to YJM789 in YM [31]. In contrast, the expression of *DIP5* increased in glyphosate treatment in YJM789 and to a lesser extent in AWRI1631 and RM11, but not S288c (Fig 6A). Growth inhibition of the other strains of yeast on exposure to glyphosate was rescued by addition of aspartic acid (Fig 6B). Additionally, the homozygous *dip5Δ* mutant, did have improved growth with the addition of WYF compared to the *DIP5*^*S288c*^ hemizygous diploid. To quantitate the ability of different amino acids to regulate the function of Dip5, quantitative growth assays were performed with two haploid S288c strains (BY4741 and GSY147). In the absence of glyphosate, deletion of *DIP5* did not change the growth of either strain, except GSY147 *dip5Δ* grew slightly slower than GSY147 in YPD (Fig 6C). Both *dip5Δ* strains grew better then wild-type controls when glyphosate was added (Fig 6C). The addition of aspartic acid downregulates Dip5 at the plasma membrane [30]. The improved growth of the *dip5* mutants on addition of aspartic acid suggests that import of glyphosate by Dip5 is a general mechanism, however there are other aspartic acid regulated modes of transport into the cell.

Because *aro1Δ* mutants are sensitive to many drugs [32], we transformed alleles of *PDR5* into both wild-type and *aro1Δ* mutants to determine if expression of *PDR5*^*S288c*^ could bypass sensitivity of *aro1Δ* in the presence of glyphosate. On YPD, both S288c and YJM789 *aro1Δ* mutants did not grow in the presence of glyphosate. Expression of each allele of *PDR5* in YJM789 *aro1Δ* decreased growth in WYF media and completely abolished growth when supplemented with glyphosate (S4 Fig). Surprisingly, the YJM789 *aro1Δ* mutant grew on WYF with glyphosate but not on YPD with glyphosate, where growth was abolished with the ectopic expression of any allele of *PDR5*.

## Discussion

When grown in minimal media, the canonical target of glyphosate, the shikimate pathway, was affected and could be rescued by supplementation of downstream metabolites in most yeast. Utilizing an existing recombinant haploid yeast collection, two different loci linked to genetic differences in glyphosate response in S288c and YJM789 yeast were identified. Expression of *PDR5*^*S288c*^ in YJM789 rescued sensitivity to glyphosate in rich media, while deletion in S288c conferred sensitivity. Pdr5 is the yeast ortholog of Mdr1, a multiple drug transporter that is highly polymorphic in yeast [15]. The second gene linked to glyphosate response was *DIP5*, which encoded an aspartic acid and glutamic acid permease [29]. The identification of these two genes furthers our understanding of the mechanisms by which glyphosate is transported in and out of the cell.

S288c is a domesticated strain of *S*. *cerevisiae* and has been the subject of extensive phenotypic, molecular and genetic analysis. Conducting studies on one genetic background limits the perspective. For example, *aro1* mutants displayed differences in viability in RM11, S288c, and YJM789 strains and their growth on nutrient limiting media. Unlike S288c, *ARO1* was essential for growth of RM11 on rich media but that was not from polymorphisms or a novel function in Aro1^RM11^ but from a synthetic lethal interaction with an unknown allele in the RM11 genome. YJM789 *aro1* mutants grew poorly on glyphosate and WYF alone, and were rescued by expressing an extra copy of *PDR5* but only in WYF, when the function of Aro1 to produce chorismate was thought to be bypassed. Despite *pdr5* mutants having multiple drug sensitivities in S288c, *aro1* mutants show no increase in growth when expressing one extra copy of *PDR5*. RNA analysis of a slightly different glyphosate formulation found genes regulating membrane stress in response to glyphosate [33]. The sensitivity to the commercial preparation of glyphosate could only be in part a response due to other additives for two reasons. The yeast growth profiles were not the same in YPD and WYF and expression of Pdr5 alleles in WYF only showed a small rescue. Previous studies did not directly identify the target of glyphosate in rich media, but protein coding polymorphisms in Pdr5^S288c^ and Pdr5^AWRI1631^ would affect export of glyphosate.

The best characterized target of glyphosate is 5-enolpyruvylshikimate-3-phosphate synthase in the shikimate pathway, and indeed all strains showed improved growth with WYF remediation. In addition, S288c and YJM789 *aro1Δ* cells grew slower on WYF with glyphosate than wild-type parental strains. This may be an indirect effect, as S288c *aro1Δ* yeast have been found to be sensitive to multiple drugs [32]. An alternative explanation is that there are other targets of glyphosate that become affected when the primary target of glyphosate is deleted. Nevertheless, the increased toxicity of glyphosate when downstream metabolites of the shikimate pathway were provided, could be an indication of the presence of non-canonical glyphosate targets. Even sensitivity to glyphosate in rich media was unexpected. The shikimate pathway is down-regulated by the presence of aromatic amino acids, and the expression of Aro1 decreased in YPD compared to YM in S288c [34]. No change in Dip5 protein levels was detected in S288c by previous studies, while Pdr5 protein levels decreased in YM compared to YPD [34]. In rich media, nutrient transport pathways are unregulated and biosynthetic pathways are down regulated. While in minimal media, the opposite effect is observed in pathway regulation and expression. Also, there may be additional glyphosate-sensitive targets and future work will address their identification. Reports have found that glyphosate chelates calcium, manganese, iron, and magnesium [35] and glyphosate-sensitive soybean have lower levels of these minerals [36]. The shikimate pathway was originally identified years after the invention of glyphosate, by identifying increased levels of shikimate in glyphosate treated plants [2], similar methods may be applied to identify other affected pathways.

Dip5 is located at the plasma membrane, and it transports aspartic acid and glutamic acid into the yeast cell. When there is excessive aspartic acid, Dip5 is targeted for endocytosis via arrestins and through ubiquitination it is targeted for degradation [30, 37]. Deletion of *DIP5* and expression of *DIP5*^*YJM789*^ further increased glyphosate tolerance of the cell. In yeast cells that expressed only the *DIP5*^*YJM789*^ allele, glyphosate resistance increased in YM, compared to yeast containing the *DIP5*^*S288c*^ allele which did not show the same. We proposed that Dip5 at the plasma membrane is at least one of the proteins involved in transporting glyphosate into the cell. This process is regulated by phosphorylations that promote ubiquitination [30], but there were no polymorphisms at any of these known residues (S3 Fig). Within the first 74 nucleotides there were three SNPs. From global transcriptomics [31] and mRNA expression it has been determined that the level of *DIP5* mRNA is two-fold lower in YJM789. As S288c (GSY147) *dip5Δ* rescue was less pronounced than BY4741 *dip5Δ*, it can be concluded that Dip5 may be differently regulated between these strains. In addition, *DIP5*^*S288c*^ may be regulated differently than *DIP5*^*YJM789*^ because there was no rescue in *DIP5*^*S288c*^ with the addition of aspartic acid to levels of glyphosate tested here. Yeast expressing *DIP5*^*YJM789*^ was not the same as the knockout, which suggests that there is an additional function of *DIP5*^*YJM789*^ compared to *DIP5*^*S288c*^. The lower levels of Dip5 in YJM789 at the membrane which will internalize glyphosate may be downregulated faster than Dip5^S288c^. The addition of glyphosate increased the amount of *DIP5* mRNAs in all the strains tested. The addition of aspartic acid rescued all strains including *dip5* mutants suggesting that there are other transporters of glyphosate.

In this study, we have uncovered one path of glyphosate import and one path of glyphosate export, and identified the differences within these transporters in the various strains. The polymorphisms in *DIP5* and *PDR5* determine the entry and pumping out of glyphosate from the cell, respectively. We propose that polymorphisms, and differences in the Dip5 protein levels, change the amount of glyphosate transported into the cell. Dip5^YJM789^ transports less glyphosate than Dip5^S288c^ and both alleles are down-regulated by the addition of aspartic acid through ubiquitination (Ub) and endocytosis. Once inside the yeast, glyphosate inhibits Aro1 and possibly other non-canonical targets. Either glyphosate or metabolized products are then transported out of the yeast by Pdr5 with the S288c allele being more active than the YJM789 allele. The allele present, in turn has a correlation with growth inhibition on exposure to glyphosate. Additional studies may reveal targets of glyphosate outside the shikimate pathway that could be classified as non-canonical targets. This study focused on differences of two yeast strains that varied in their glyphosate transport pathways. With the widespread use of glyphosate, encroachment of developments into pristine areas, and the efforts to control weeds and invasive species in state parks, glyphosate resistance is likely to continue its spread in the wild.

## Materials & methods

### Yeast strains and plasmids

Previously published strains and their derivatives are in S1 Table. *ARO1*, *DIP5 and PDR5* were knocked out using homologous recombination with the dominant drug resistance Nat^R^ or Kan^R^ as previously described [38] and listed in S1 Table. In YJM789, *DIP5* was replaced with *dip5*::*KanR* using BY4741 dip5 as the template and the following primers 5’ AAA GTA CCA CAT ATC TAA CG 3’ and 5’ GTG ATA CCT GTA CAC TAT GGT TCC 3’. Cloning of *ARO1* alleles was done by PCR amplified from genomic DNA using primers as follows 5’ARO1 5’ATG ACC ATG ATT ACG CCA AGC TTG CAT GCC TGC AGG TCG AGC CAA TCT CAC AGA TTT AAT ATA G3’, 3’ARO1 5’TAT ATT GAT CAC CGA TAT ATG GAC TTC CAC ACC AAC TAG TAA TTC TTC AGT GAA TAA ACG GGC C3’, 5’PDR5 5’GAT TAC GCC AAG CTT GCA TGC CTG CAG GTC GAC TCT AGA CTA ATC CAA TTC AGT TGT CTC3’, and 3’PDR5 5’ATC ACC GAT ATA TGG ACT TCC ACA CCA ACT AGT TTC GGA CAG ATA ATG ATA TAA TAT ATC3’. All cloned genes were accompanied by their surrounding intergenic sequences up to the neighboring upstream and downstream genes. Genes were cloned via homologous recombination into the XbaI and SpeI sites of pGS36 plasmid with hygromycin resistance [39]. Plasmids were kept under selection with hygromycin after LiAc chemical transformations [40]. *ARO1* was knocked out with a PCR cassette containing Nat^R^ in YJM789K5a, GSY147, and RM11 MATa/MATα and plated onto YPD containing 1μg/ml nourseothricin. RM11 MATα, *ho*::*Kan*^*R*^ was crossed to GSY147 MATa, *aro1*:*Nat*^*R*^ and selected on YM with nourseothricin and G418. RM11 *aro1Δ/ ARO1* diploid carrying plasmids expressing *ARO1* were sporulated and dissected onto YPD with hygromycin to select for the plasmid. There was genetic variation in tolerance of strains to hygromycin in YM. RM11 strains grown in YM required twice as much hygromycin (250 μg/ml) as required in YPD to maintain plasmids under hygromycin selection. Hemizygous hybrid yeast strains were constructed by transforming the wild-type parent with pGS36 and mating it with the mutant parent. Hemizygous yeast were selected with hygromycin and nourseothricin or G418 depending on the dominant selectable markers. The respective hemizygous genotypes and their markers are listed in S1 Table. The haploid recombinant segregant collection between YJM789 and S288c (S96) was previously generated [23].

### Media and chemicals

All yeast strains were grown in nutrient rich media (YPD) or minimal media (YM) which includes 2% dextrose, 6.7g/L yeast nitrogen base and 20g/L agar in solid media. WYF plates contained 20 μg/ml tryptophan, 30 μg/ ml tyrosine and 50 μg/ ml phenylalanine added to YM plates while D plates were supplemented with 100 μg/ml aspartic acid. Plates lacking *para*-Aminobenzoic acid (PABA) were made from yeast nitrogen base lacking PABA (Sunrise^®^). Credit^®^ 41 Extra contained 41% glyphosate and surfactants. The haploid recombinant segregant collection contained *lys2* and *lys5* alleles segregating in the cross and therefore, lysine was added to media for these strains. YM and WYF media were also supplemented with histidine, uracil, leucine, and methionine for BY4741 strains. Yeast were grown overnight to saturation and then diluted to 0.1 OD units (approximately 1X10^7^ cells) and serially diluted 10-fold. Dilutions were then stamped on to plates. Growth was scored on solid media after 2–3 days of growth, relative to a control without glyphosate to account for how many of the spots grew. Quantitative growth assays were carried out in a TECAN automatic plate reader as previously described [41].

### RNA quantitation

Total RNA was isolated by hot phenol extraction [42] and precipitated from cultures grown to mid-log phase and treated for 90 minutes with 0.25% glyphosate. RNA treated with DNAse I was then converted to cDNA using Invitrogen SuperScript^®^ III First-Strand kit according to the manufacturer’s directions. cDNA from biological duplicates was amplified in triplicate using specific primers in SSO FAST on a BioRad real-time PCR system. *ARO1* primers used were upstream 5’ACCGACTGGTTAGGTATCCG3’ and downstream 5’CCTAAACTGTGCAAGGCGTA 3’. 25S rRNA and genomic DNA were used to normalize samples with the following primers upstream 5’GACTACTTGCGTGCCTTGTTG3’ and downstream 5’CCGTTCCCTTGGCTGTG3’. *PDR5* primers used were 5’GTT GGC TGT TGG TGT TGC TA3’ and downstream 5’AAC TAC AGG TGT CAG TGG CA3’.

*DIP5* primers used were upstream 5’CTG CTG CTT TGG TCA TTC AA3’ and downstream 5’ TGG TTA GGA CCT CCA CCA AG3’.

### Mapping genetic linkage

Quantitative trait loci (QTL) mapping for S96 x YJM789 haploid segregants in three different conditions was carried out as previously described [43]. The statistical threshold for each trait was calculated independently with 1000 permutations using R package qtl with EM method. Genes and coordinates under the peak of association for each condition was referenced using *Saccharomyces* Genome Database [44]. QTL scores are listed in S2–S4 Tables.

## Supporting information

S1 Fig Aro1 is a pentafunctional enzyme in the shikimate pathway. **A**. Schematic of enzyme functions in Aro1 with bacterial proteins AroB (3-dehydroquinate synthase amino acids 1–392), AroA (EPSPS amino acids 404–861), AroL (shikimate kinase amino acids 887–1060), AroD (3-dehydroquinase amino acids 1061–1295), and AroE (shikimate dehydrogenase amino acids 1306–1599). **B**. Alignment of the ESPS glyphosate binding site across different species. In red are residues that when mutated confer resistance to glyphosate in *E*. *coli*. **C**. RNA expression levels of *ARO1* mRNA from RM11, YJM789 and YJM789 *aro1Δ* carrying different alleles *ARO1* grown in YM with and without 0.25% glyphosate. Q RT-PCR mRNA of *ARO1* levels are normalized to 25S rRNA. **D**. Tetrad dissections of RM11 heterozygous knockout of *ARO1* compared to wild-type RM11 diploid on YPD. Tetrads were numbered and haploid segregant germinating from a single spore are lettered. Plates were incubated at 30°C for two days. **E**. Tetrad dissections of RM11 wildtype and S288c *aro1Δ* hybrids (F1) were incubated for five days before being photographed. Haploid segregants from F1 yeast with *aro1Δ* were circled.
(TIF)

S2 Fig N-rooted phylogenetic tree of Pdr5 protein from genetically different yeast. Branch length was determined by UPGMA in ClustalW. Relative growth of yeast on glyphosate was normalized to growth with no treatment.
(TIF)

S3 Fig N-rooted phylogenetic tree of Dip5 protein from genetically different yeast. Branch length was determined by UPGMA in ClustalW. Relative growth of yeast on glyphosate was normalized to growth with no treatment.
(TIF)

S4 Fig Ectopic expression of *PDR5* alleles in S288c and YJM789 with and without *ARO1*. *ARO1* was knocked out in S288c and YJM789. *PDR5* was cloned and expressed from its native promoter from a plasmid. Yeast were grown on YPD (rich media) with 1% glyphosate, YM (minimal media) with 0.25% glyphosate and WYF (yeast minimal media supplemented with aromatic amino acids) with 0.25% glyphosate.
(TIF)

S1 Table List of yeast used in this study. (XLSX)

S2 Table QTL mapping between S288c and YJM789 on 1% glyphosate on YPD. (XLSX)

S3 Table QTL mapping between S288c and YJM789 on 0.15% glyphosate on WYF. (XLSX)

S4 Table QTL mapping between S288c and YJM789 on 0.15% glyphosate on YM. (XLSX)
