# Supplementary figures and images for: Genetic variation in Dip5, an amino acid permease, and Pdr5, a multiple drug transporter, regulates glyphosate resistance in S. cerevisiae
Source: PLoS One. 2017 Nov 20;12(11):e0187522. doi: 10.1371/journal.pone.0187522 (PMC5695762; doi:10.1371/journal.pone.0187522)

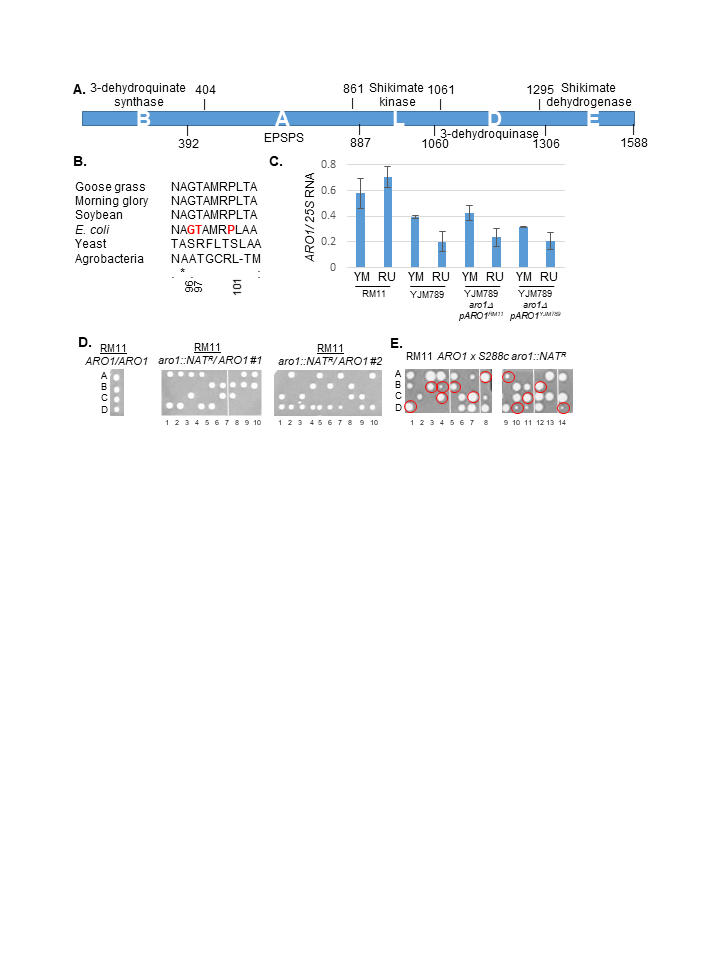

Supplement: S1 Fig — A. Schematic of enzyme functions in Aro1 with bacterial proteins AroB (3-dehydroquinate synthase amino acids 1–392), AroA (EPSPS amino acids 404–861), AroL (shikimate kinase amino acids 887–1060), AroD (3-dehydroquinase amino acids 1061–1295), and AroE (shikimate dehydrogenase amino acids 1306–1599). B. Alignment of the ESPS glyphosate binding site across different species. In red are residues that when mutated confer resistance to glyphosate in E. coli. C. RNA expression levels of ARO1 mRNA from RM11, YJM789 and YJM789 aro1Δ carrying different alleles ARO1 grown in YM with and without 0.25% glyphosate. Q RT-PCR mRNA of ARO1 levels are normalized to 25S rRNA. D. Tetrad dissections of RM11 heterozygous knockout of ARO1 compared to wild-type RM11 diploid on YPD. Tetrads were numbered and haploid segregant germinating from a single spore are lettered. Plates were incubated at 30°C for two days. E. Tetrad dissections of RM11 wildtype and S288c aro1Δ hybrids (F1) were incubated for five days before being photographed. Haploid segregants from F1 yeast with aro1Δ were circled. (TIF) [file pone.0187522.s001.TIF]

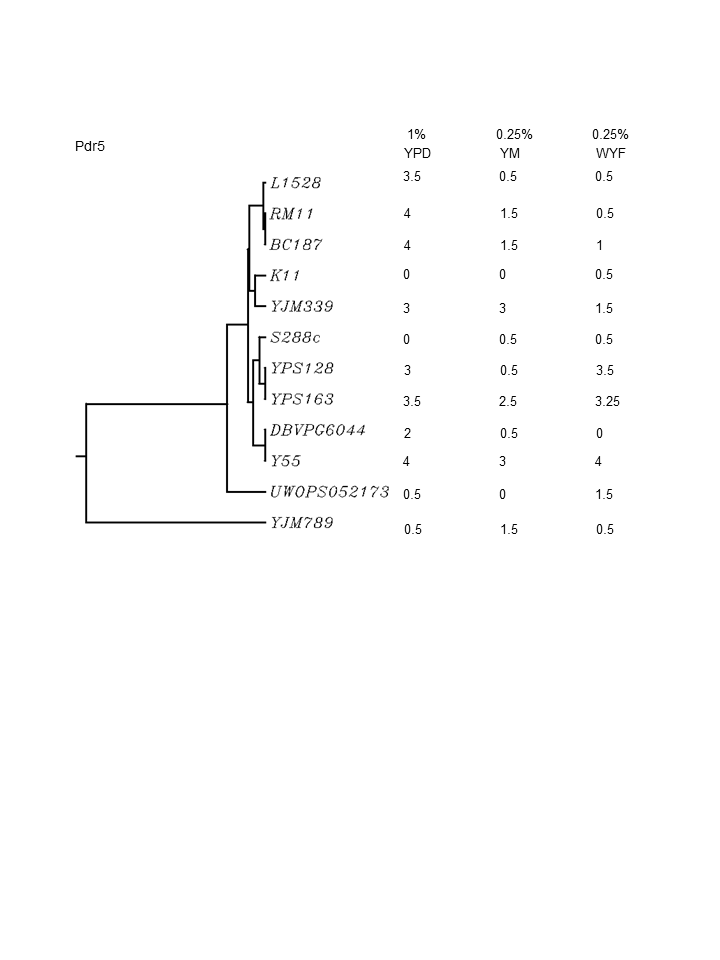

Supplement: S2 Fig — Branch length was determined by UPGMA in ClustalW. Relative growth of yeast on glyphosate was normalized to growth with no treatment. (TIF) [file pone.0187522.s002.TIF]

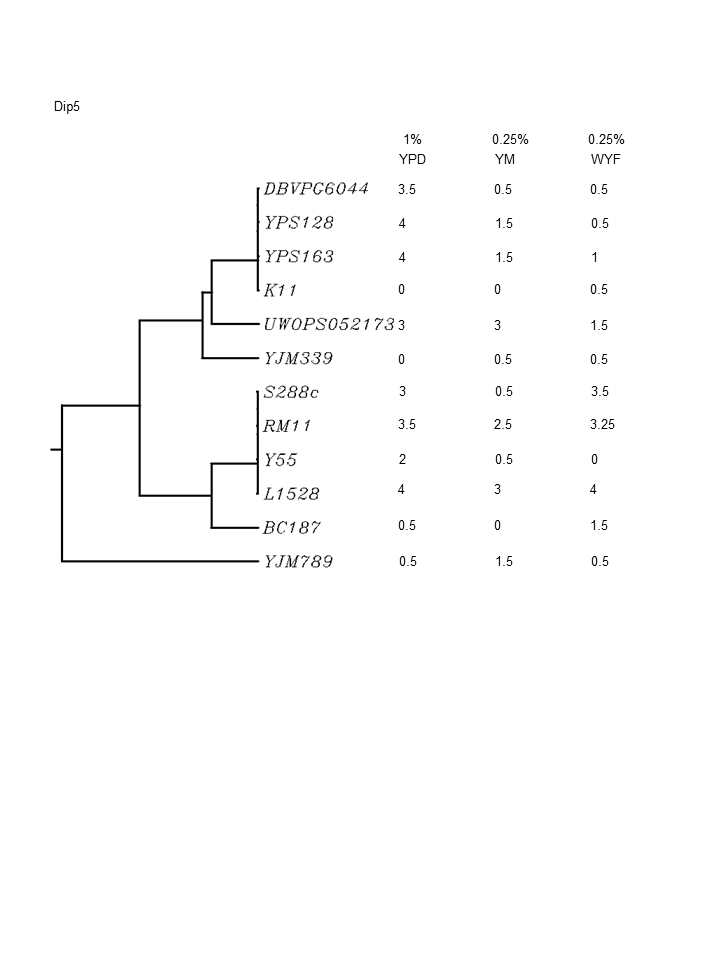

Supplement: S3 Fig — Branch length was determined by UPGMA in ClustalW. Relative growth of yeast on glyphosate was normalized to growth with no treatment. (TIF) [file pone.0187522.s003.TIF]

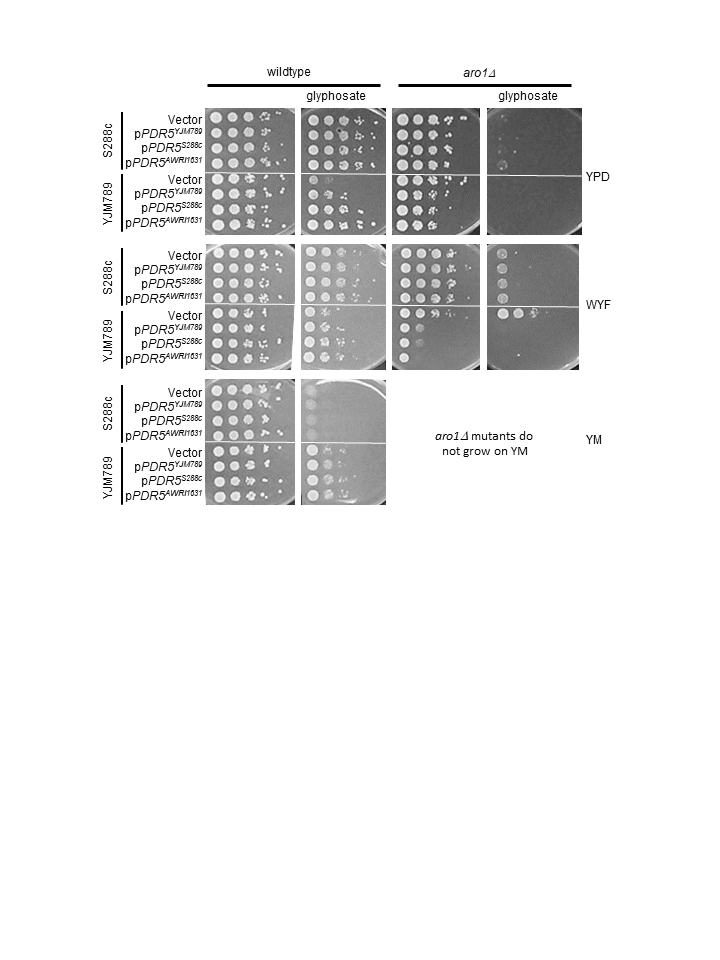

Supplement: S4 Fig — ARO1 was knocked out in S288c and YJM789. PDR5 was cloned and expressed from its native promoter from a plasmid. Yeast were grown on YPD (rich media) with 1% glyphosate, YM (minimal media) with 0.25% glyphosate and WYF (yeast minimal media supplemented with aromatic amino acids) with 0.25% glyphosate. (TIF) [file pone.0187522.s004.TIF]
